# 5-aminolevulinic enhanced brain lesions mimic glioblastoma: A case report and literature review

**DOI:** 10.1097/MD.0000000000034518

**Published:** 2024-01-05

**Authors:** Chao-Yuan Chang, Chun-Chung Chen

**Affiliations:** a Neurosurgical Department, China Medical University Hospital, Taichung, Taiwan; b Department of Surgery, College of Medicine, China Medical University, Taichung, Taiwan.

**Keywords:** 5-aminolevulinic acid, fluorescence-guided surgery, glioblastoma multiforme

## Abstract

**Rationale:**

Glioblastoma multiforme (GBM) is a highly malignant primary brain tumor for which maximal tumor resection plays an important role in the treatment strategy. 5-aminolevulinic (5-ALA) is a powerful tool in fluorescence-guided surgery for GBM. However, 5-ALA- enhancing lesion can also be observed with different etiologies.

**Patients concerns:**

Three cases of 5-ALA-enhancing lesions with etiologies different from glioma

**Diagnoses:**

The final diagnosis was abscess in 1 patient and diffuse large B-cell in the other 2 patients.

**Interventions:**

Three patients received 5-aminolevulinic acid-guided tumor resection under microscope with intraoperative neuromonitoring.

**Outcomes:**

All of our patients showed improvement or stable neurological function outcomes. The final pathology revealed etiologies different from GBM.

**Lessons:**

The 5-aminolevulinic acid fluorescence-guided surgery has demonstrated its maximal extent of resection and safety profile in patients with high-grade glioma. Non-glioma etiologies may also mimic GBM in 5-ALA-guided surgeries. Therefore, patient history taking and consideration of brain images are necessary for the interpretation of 5-ALA-enhanced lesions.

## 1. Introduction

Glioblastoma multiforme (GBM) is a highly malignant primary brain tumor for which maximal tumor resection plays an important role in the treatment strategy. Advancements in navigation systems, intraoperative neuromonitoring, and fluorescence-guided surgery (FGS) have facilitated maximal tumor burden reduction and preservation of neurological function.

FGS is an adjunctive operative method that depends on different fluorophores administered and accumulated in the target lesion. FDA-approved fluorophores, including indocyanine green and fluorescein, have long-term utilization that assists in lesion resection, especially in oncological surgery.

High-grade gliomas usually exhibit an infiltrative pattern that is difficult to discriminate from normal brain tissues. 5-aminolevulinic acid (5-ALA, Gliolan, Medac, Wedel, Germany) is a precursor of porphyrin that accumulates in malignant glioma cells and emits intense pink fluorescence intraoperatively under the BLUE-400 filter (Pentero, Carl Zeiss Meditech, Oberkochen, Germany).

Over the long-term, 5-ALA is a powerful tool in FGS for GBM surgery, which leads to complete resection and more than 6 months of progression-free survival. Radiographically, on T1-weighted magnetic resonance imaging (MRI) with a gadolinium enhancement sequence, the GBM can manifest as a heterogeneously enhancing lesion with profound perifocal edema. These characteristics can also be observed in other types of brain lesions. In this report, we present 3 5-ALA-enhanced cases that mimic GBM, and review the literature.

## 2. Cases

### 2.1. Clinical presentation: Case 1

**Figure 1.**
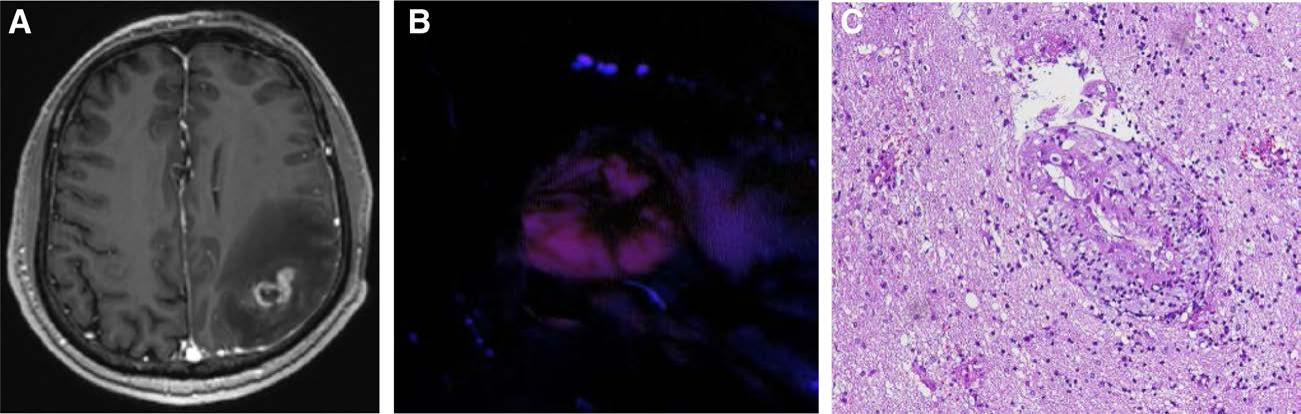
(A) Patient 1 preoperative images. T1-weighted magnetic resonance with contrast image disclosed right parietal intra-axial ring-enhancing lesion with perifocal edema. (B) Patient 1 intraoperative image under blue-400 filter revealed strong 5-ALA-enhanced lesion. (C) Patient 1 pathological report revealed vascular proliferation, perivascular lymphoplasmacytic cuffing, abscess formation, and peri-meningeal fibrosis with focal granulation tissue formation, as assessed by hematoxylin and eosin staining. 5-ALA = 5-aminolevulinic.

A 59-year-old male with a high educational level and no significant comorbidities complained of occasional difficulty in word recognition for 1 month. One of his daily hobbies is gardening. He suffered acute right-sided weakness and progressive slurred speech for 1 day. On initial examination, the patient was well oriented. The Glasgow coma scale (GCS) revealed E4V5M6, and the muscle strength grading of the right limbs was 4+. The white blood cell count and C-reactive protein level were within the normal range, with no elevation of tumor markers detected on blood examination. Brain computed tomography and MRI with gadolinium (Gd) enhancement scans disclosed a right parietal intra-axial ring-enhancing lesion (30 mm × 25 mm × 16 mm) with “finger-like” white matter edema (Fig. 1A). Based on these findings, high-grade glioma was highly suspected as the first diagnosis, followed by metastasis, abscess, or lymphoma. 5-aminolevulinic acid-guided brain tumor resection surgery was performed. Intraoperatively, the tumor showed vivid enhancement with 5-ALA (Fig. 1B), but the frozen section demonstrated no malignancy. The pathological report revealed vascular proliferation, perivascular lymphoplasmacytic cuffing and abscess formation (Fig. 1C). The final specimen culture report showed the presence of Nocardia species. Transient aphasia and fluctuations in right muscle power were noted postoperatively. After 4 weeks of empirical imipenem, trimethoprim, and sulfamethoxazole treatment, his muscle power and language function improved, and discharge proceeded uneventfully.

### 2.2. Clinical presentation: Case 2

**Figure 2.**
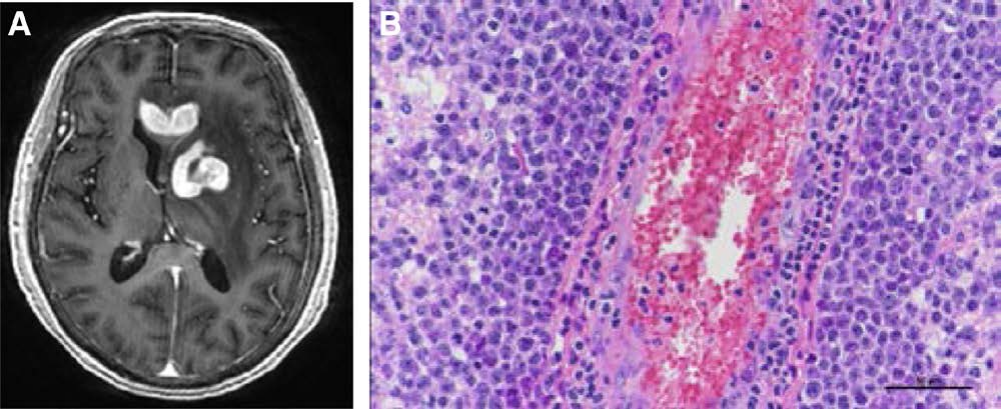
(A) Patient 2 preoperative image. T1-weighted magnetic resonance with contrast image disclosed heterogeneously enhanced lesion over corpus callosum and left basal ganglion with perifocal edema. (B) Patient 2 pathology report revealed cerebral tissues with perivascular and diffuse infiltration of large atypical lymphoid cells with nuclear pleomorphism, vesicular nuclei, prominent nucleoli, and brisk mitotic activity. Hematoxylin and eosin staining demonstrated diffuse large B-cell lymphoma of non-GC type.

A 73-year-old female with hypertension and diabetes mellitus complained of headache and dizziness, Her GCS was E4V5M6, she was well oriented, and muscle grading of the 4 extremities was full. The complete blood count and biochemical examination results were unremarkable. Chest and abdominal imaging revealed a negative primary malignancy. Brain computed tomography and MRI with Gd + revealed heterogeneously enhanced lesions in the corpus callosum and left basal ganglion (Fig. 2A). Based on these findings, glioblastoma and lymphoma were considered the most probable diagnoses. 5-aminolevulinic acid-guided tumor resection with intraoperative neuromonitoring was performed. Intraoperatively, the brain lesion showed strong 5-ALA-guided enhancement, whereas the frozen section could not distinguish lymphoma and glioblastoma. A neurological preservation surgery was planned. The final pathological showed cerebral tissues with perivascular and diffuse infiltration of large atypical lymphoid cells with nuclear pleomorphism (Fig. 2B). Diffuse large B-cell lymphoma was diagnosed. The bone marrow biopsy results were negative, suggesting no bone marrow involvement. The patient was discharged uneventfully with no new neurological defects and was transferred to the hematology and oncology department for continued treatment.

### 2.3. Clinical presentation: Case 3

**Figure 3.**
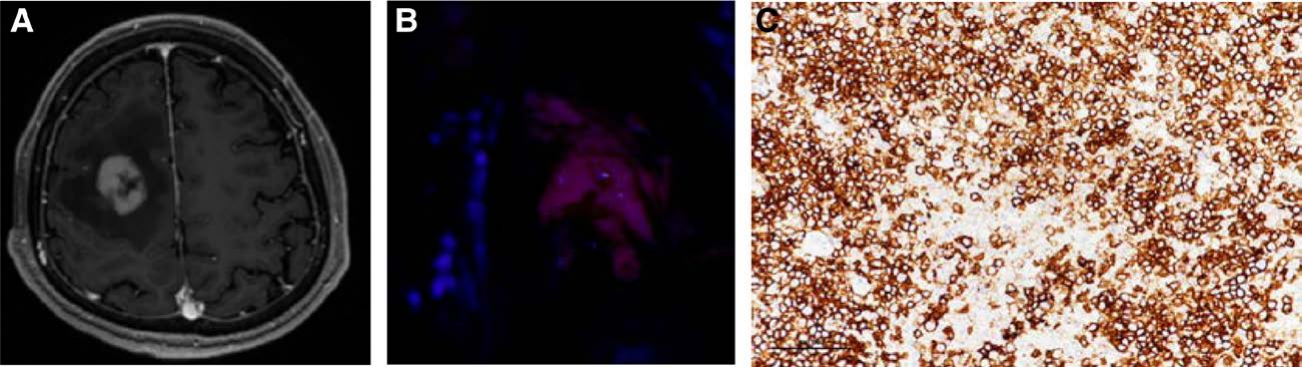
(A) Patient 3 preoperative image. T1-weighted magnetic resonance with contrast image revealed a vivid enhancing lesion over right frontal-parietal area. (B) Intraoperative microscopic view under blue-400 filter revealed strong 5-ALA-enhanced lesion. (C) Immunostaining with CD20 demonstrating tumor cells with a B-cell immune phenotype. 5-ALA = 5-aminolevulinic.

Here, we reported a 65 years old male with a history of hypertension. He had left-sided weakness for approximately 1 week. His GCS score was E4V5M6 and the muscle grading of the left side was 4+. Magnetic resonance imaging with Gd + demonstrated a vivid enhancing lesion, measuring 30 mm × 21 mm × 26 mm, in the right parietal lobe with prominent perifocal edema (Fig. 3A). A highly malignant primary tumor was suspected. A preoperative systemic survey excluded brain lesion metastases from other organs. 5-aminolevulinic acid-guided microscopic surgery was planned with continuous intraoperative electrophysiological monitoring, because the lesion was located near the eloquent area. Intraoperatively, the suspicious tissue showed obvious fluorescence under a blue-400 excitation wavelength (Fig. 3B). Frozen resection was positive for malignancy, but there was no conclusive recognition of the lesion as a glioma or lymphoma lineage. The tumor was completely resected. The patient experienced transient left-sided weakness. The immunostaining with CD20 demonstrating tumor cells with a B-cell immune phenotype (Fig. 3C), a permanent pathological report revealed diffuse large B-cell lymphoma.

## 3. Different fluorescent dyes used in brain surgery (Table 1)

### 3.1. Fluorescein

Fluorescein sodium has been used in brain tumor surgery, for example, in glioma, meningioma, and metastasis,^[4,21–23]^ whereas fluorescein is used in intracranial aneurysm clipping surgery.^[23]^ Known as an FDA-approved fluorophore that is used in ocular angiography, fluorescein can form a complex with proteins in the bloodstream and accumulate in the tumor where the blood-brain barrier is disrupted. Owing to the small molecular weight of fluorescein as compared to that of gadolinium, the extent of enhanced-region target lesion under microscope is usually larger than gadolinium-enhanced lesion on MR. Under the YELLOW 560-filter on the Pentero microscope (Carl Zeiss Meditec, Germany), the targeted lesion can be vividly enhanced to help distinguish it from normal brain tissue. Fluorescein-guided surgery in GBM patients showed improved GTR (83%) compared to that of the group with not fluorescein use (55%).^[7,24]^ Owing to the infiltrative nature of glioma, conventional gadolinium-enhanced MRI may underestimate the actual tumor size, and neurosurgeons often take T2-weighted and fluid-attenuated inversion recovery images into consideration for preoperative tumor resection plan. Intraoperative cerebrospinal fluid drainage and brain shifting after dural incision may cause the navigation system to lose its accuracy. Fluorescein is beneficial in guiding tumor resection without obvious side effects. However, surgical manipulation of the brain tissue may also cause normal blood-brain barrier disruption and normal brain tissue enhancement. Therefore, meticulous techniques are crucial for distinguishing normal from pathological brain tissues.

**Table 1:** Fluorophores for applications in high-grade glioma surgery.

Fluorophore	Used equipment	Principle	Administration/time consuming	Advantages	Disadvantages	Reference
5-Aminolevulinic acid	• Zeiss Pentero Microscope (Carl Zeiss Surgical GmbH	• Metabolism, Disruption of BBB necessary for fluorophore accumulation	• IV, 2 h	• Safety profile • Selectively absorbed by tumor cells • Intraoperative real-time imaging	• Depth can limit visualization • Low fluorescence intensity in low-grade gliomas • Requires special microscope • Expensive • Time dependent	• ^[1–3]^
Fluorescein	• Zeiss Pentero Microscope (Carl Zeiss Surgical GmbH) • LSM710 (Carl Zeiss Surgical, GmbH)	• Permeability of BBB	• IV, 5 min	• Safety profile • Intraoperative real-time imaging • Can be visualize under white light (using higher concentrations)	• Not specifically absorbed by tumor cell • Possible extravasation along with edema or surgical procedure • Time dependent	• ^[4–7]^
ICG	• Zeiss Pentero Microscope	• Permeability of BBB	• IV, 24 h	• Enables visualization of fluorescence situated deeper in tissue • Low toxicity, high safety • Enables visualization of fluorescence situated deeper in tissue	• Accumulates because of enhanced permeability of BBB • Special camera is needed • Time dependent	• ^[8–10]^
BLZ-100 (tozuleristide)	• FLUOBEAMR 80, 023 (Fluoptics, Grenoble, France) and the synchronized infrared imaging system (SIRIS)	• Tumor-specific	• IV, 48 h	• High affinity to human gliomas	• Needs more clinical studies to prove safety and efficacy	• ^[11–15]^
Tumor-targeted alkylphosphocholine analogues	• CLR1501: Nikon A1RSi confocal microscope • CLR1502: Leica OH4 intraoperative microscope with FL800 attachment	• Tumor-specific	• IV, 4 d	• CLR1501, Tumor-to-brain fluorescence ratio similar to 5-ALA • CLR1502, Tumor-to-brain fluorescence ratio superior to 5-ALA	• Tumor must be visualized on separate monitor	• ^[16–18]^
Cetuximab-IRDye 800	• Wide-field optical imaging system PINPOINT (Novadaq, Burnaby, Canada) • Wide-field surg vision explorer air (surg vision BV, ‘t Harde, The Netherlands)	• Tumor-specific	• IV, 48 h	• Specific detection of tumor cells	• Needs more clinical studies to prove safety and efficacy	• ^[19,20]^

Modified by Fluorescence-guided Brain Tumor Surgery, Youmans and Winn Neurological Surgery, Eighth Edition.

5-ALA = 5-aminolevulinic, ICG = indocyanine green.

### 3.2. Indocyanine green

Indocyanine green (ICG) is an amphiphilic molecule used in cardiovascular, liver, and ophthalmic angiographies.^[25]^ Since 2003, ICG has gradually played an adjuvant role in the neurosurgery fields, including aneurysm and arteriovenous malformation surgery.^[9,26,27]^ At the excitation and emission peaks of 805 nm and 835 nm respectively, ICG can be taken up at 0.2 to 0.5 mg/kg in 1 minute for video angiography.^[9,28]^ Higher doses of ICG up to 5 mg/kg can be intravenously administrated 16 and 30 hours prior to brain tumor surgery, in 1 hour duration. This novel technique, known as second-window ICG, differs from the traditional technique.^[10,29]^ Owing to its near-infrared characteristics, it can penetrate tissues of up to 15 mm in diameter. Enhanced permeability and retention due to blood-brain barrier breakdown, impaired lymphatic drainage, and permeability mediators increases ICG accumulation surrounding the brain tumor tissue. The published second-window ICG-assisted brain tumor studies included gliomas,^[10]^ meningiomas,^[30]^ metastases,^[31]^ pituitary macroadenomas,^[32]^ and intraventricular tumor.^[33]^ No major complications have been reported after administering ICG as an adjuvant during brain tumor surgery.^[27]^

### 3.3. BLZ-100 (tozuleristide)

Tozuleristide is a conjugate of CTX and ICG. Chlorotoxin is a 36-amino acid peptide that was initially isolated from scorpion venom. All clinical trials have demonstrated that CTX exhibits negligible toxicity in humans. It can specifically bind tumors via a molecular interaction with Annexin A2 and matrix metalloproteinase 2.^[34]^ Conjugated with ICG, BLZ-100 (tozuleristide or Tumor Paint) was demonstrated to be a tumor-bound agent that can be used in high- and low-grade glioma, for example, GBM, astrocytoma, ependymoma, and meningioma in vitro.^[14,15]^ In a phase I human clinical study, BLZ-100 demonstrated its greater fluorescence intensity in high-grade glioma as compared to low-grade glioma. In addition, the fluorescence signal intensity increased with increasing dose of BLZ-100. This agent can be injected as a single dose from 3 mg to 30 mg and can be detected in the operation as early as 3 hours after administration.^[11]^ BLZ-100 combines the advantages of a tumor-specific-targeted peptide and near-infrared (NIR) technique that can penetrate a tissue depth of approximately 15 mm, and its safety profile has shown that it is well tolerated.^[32,33]^

### 3.4. Tumor-targeted alkylphosphocholine analogues

Alkylphosphocholine analogs are synthetic molecules that can be used in tumor-selective positron emission tomography imaging, ^124^I-CLR1404 therapeutic radiation,^[14,15,34]^ or as a kind of “diapeutic” (diagnostic imaging and therapy) agent. Alkylphosphocholine.

analogs have a cancer-selective mechanism and can predict, inform, and monitor therapeutic outcomes.^[35]^ Two of the ^124^I-CLR1404 derivatives, CLR1501 (green) and CRL1502 (near-infrared), demonstrated specific affinity towards GBM in in vitro and in vivo studies,^[18]^ 5-aminolevulinic acid and CLR1501 had similar T:N (tumor: normal brain tissue) fluorescence ratios, whereas CLR1502 had a higher T:N fluorescence ratio than 5-ALA and provided excellent tumor visualization.^[14]^

### 3.5. Cetuximab-IRDye 800

Epidermal growth factor receptor is overexpressed or mutated in 40-70% of GBM. Recent studies have demonstrated specific cancer imaging for FGS using NIR dyes conjugated to an FDA-approved monoclonal antibody for several cancer types. Cetuximab, an Epidermal growth factor receptor monoclonal antibody conjugated with IRDye800, successfully localizes to tumors in orthotopic animal models of GBM under NIR image system.^[19,36]^ The first-in-human study was published in 2018, which showed the potential to maximize the extent of primary tumor resection and to identify residual tumors.^[20]^ However, its application requires additional clinical studies. Nevertheless, there have been no reports on the severe adverse effects of cetuximab-IRDye 800.

## 4. Discussion

### 4.1. Five-ALA enhancement in high-grade glioma, low-grade glioma, and other etiologies

There are several studies on 5-ALA application in non-glioma lesions.^[37]^ A single-center retrospective study of 5-ALA used in non-glioma tumors such as brain metastasis, meningioma,^[38]^ and pituitary adenoma has been reported. The 5-ALA positive rate was 88.4% (122/138) in high-grade glioma, 77.3% (85/110) in meningioma, 52.3% (34/65) in metastasis, and 8.3% (1/12) in pituitary adenoma.^[39]^ Clinically, it is difficult to precisely distinguish glioma from other etiologies based on pre operative MR images and clinical manifestations. Although brain abscesses show characteristic-restricted diffusion in the DWI sequence of MR, there are also some case reports of brain abscesses^[40]^ that mimic GBM, *such as Aggregatibacter* and *Cryptococcus gattii*.^[41,42]^ Exogenous 5-ALA has the potential to cause accumulation of PpIX, a precursor of heme, in all rapidly dividing cells. Diffuse large B-cell lymphoma, one of the predominant lymphomas, also showed typical images similar to GBM.^[43,44]^ Though diffuse large B-cell lymphoma is a chemotherapy-sensitive disease, specific subgroups of solitary, primary, and central nervous system lymphomas may gain survival benefits in operational resection as compared to biopsy alone. As per a recently published study.^[22]^ The uptake and metabolism within lymphoma cells are poorly understood.^[45,46]^ A proton-coupled folate transporter has been declared to operate as a transporter of 5-ALA in lymphoma tissue.^[47,48]^ The positive 5-ALA induced fluorescence was noted only in 70% to 80% of primary and central nervous system lymphomas patients.^[49,50]^ Post- ischemia cerebrovascular tissues may present as GBM in the light of T1 MR with contrast images in the subacute stages, and accompanying deteriorating neurological symptoms may also indicate high-grade glioma.^[51]^ Five-ALA accumulation may lead to peritumoral edema and reactive astrocytic infiltration. These phenomena can also be observed in cases of radiation necrosis and neurodegenerative demyelinating disease.^[52]^

5-aminolevulinic acid was first approved as Gliolan (medacGmbH, Wedel, Germany) in the European Union in 2007 and has been used for neurosurgery for more than 10 years. In 2017, it was approved by the United States Food and Drug Administration. Owing to the locally disrupted blood-brain barrier and increased membrane transport and uptake by high-grade glioma cells, 5-ALA is naturally converted to PPIX.^[21,53]^ Traditionally, under violet-blue (wavelength 370–440 nm) light illumination, PPIX emits red fluorescence (635 nm and 704 nm) and guides intraoperative resection.^[2,26]^ Comparing the tumor margin between FGS using 5-ALA and Gd + MRI, FGS using 5-ALA went beyond the borders of Gd + MRI.^[54]^ One theory is that Gd + on MRI represents the disrupted blood-brain barrier area, whereas 5-ALA is metabolized specifically by tumor cells and does not completely correlate with blood-brain barrier breakdown. The 5-ALA application in the low-grade gliomas seems limited by low fluorescence visibility under the microscope, with a positive fluorescence rate ranging between 0 and 20 %.^[55,56]^ Owing to the heterogeneity of glioma, the anaplastic foci within low-grade glioma, with no evidence of preoperative contrast enhancement on MR, could be detected intraoperatively. However, the general uptake of 5-ALA preoperatively in patients with low-grade glioma needs further studies for evaluation.^[47,54,57,58]^ The 5-ALA application in a series of 12 patients with low-grade glioma showed that fluorescence emissions are very often below the detection threshold of current visual fluorescence imaging methods.^[59]^

5-aminolevulinic acid-guided microscopic surgery has proven to be an effective way to boost the resection rate in high-grade glioma cells and improve progression-free survival by 6 months, with no decline in performance status compared with conventional surgery.^[1,58]^ Recently, a prospective and multicenter study enrolling 69 high-grade glioma patients taking 5-ALA showed complete resection of enhanced tumors in 51.9% of patients, with a positive predictive value of 95.4% for high-grade glioma histopathology and diagnostic accuracy of 92.4%. Drug-related adverse events of 5-ALA occurred at a rate of 22%, including elevated liver function tests, rash, fatigue, and headache. These studies also investigated serious neurological complications following 5-ALA FGS for high-grade glioma. Consequently, newly developed neurological deficits or functional decline may occur if aggressive resection is performed in or near the eloquent area.^[60]^ However, results show that 5-ALA FGS is not associated with aggressive resections.^[56]^ Intraoperative hypotension associated with 5-ALA in malignant glioma surgery has also been reported; the risk factors were old age and intake of oral renin-angiotensin system inhibitors. The hypotension phenomenon induced by 5-ALA may be mediated by an increase in NO in vascular endothelial cells.^[59]^ According to FDA-approved regulations, 5-ALA FGS is usually performed within 2 to 4 hours of oral administration. However, a recent study demonstrated that 5-ALA-guided high-grade glioma resection can also be performed safely for more than 4 hours after administration, with clinical results largely similar to those of previous studies. However, the exact timing of fluorescence clearance from the tumor bed is currently unknown.^[61]^

## 5. Conclusion

5-aminolevulinic acid FGS has demonstrated its maximal extent of resection and safety profile in high-grade glioma. Non-glioma etiologies may also mimic GBM in 5-ALA guided surgery. Therefore, studying patient history and brain images is necessary for interpreting 5-ALA-enhanced lesions. However, the application of 5-ALA in other etiologies requires further investigation.

## Acknowledgments

The authors confirm that no governmental, institutional, or industrial funds or grants were given for the material of this study or to support any of the authors.

## Author contributions

**Conceptualization:** Chun-Chung Chen.

**Resources:** Chao-Yuan Chang.

**Supervision:** Chun-Chung Chen.

**Writing – original draft:** Chao-Yuan Chang, Chun-Chung Chen.
